# Mapping of *A*_1 _conferring resistance to the aphid *Amphorophora idaei *and *dw *(dwarfing habit) in red raspberry (*Rubus idaeus *L.) using AFLP and microsatellite markers

**DOI:** 10.1186/1471-2229-7-15

**Published:** 2007-03-20

**Authors:** Daniel J Sargent, Felicidad Fernández-Fernández, Alicja Rys, Victoria H Knight, David W Simpson, Kenneth R Tobutt

**Affiliations:** 1 East Malling Research (EMR), New Road, East Malling, Kent ME19 6BJ, UK

## Abstract

**Background:**

Raspberry breeding programmes worldwide aim to produce improved cultivars to satisfy market demands and within these programmes there are many targets, including increased fruit quality, yield and season, and improved pest and disease resistance and plant habit. The large raspberry aphid, *Amphorophora idaei*, transmits four viruses and vector resistance is an objective in raspberry breeding. The development of molecular tools that discriminate between aphid resistance genes from different sources will allow the pyramiding of such genes and the development of raspberry varieties with superior pest resistance. We have raised a red raspberry (*Rubus idaeus*) F_1 _progeny from the cross 'Malling Jewel' × 'Malling Orion' (MJ × MO), which segregates for resistance to biotype 1 of the aphid *Amphorophora idaei *and for a second phenotypic trait, dwarf habit. These traits are controlled by single genes, denoted (*A*_1_) and (*dw*) respectively.

**Results:**

The progeny of 94 seedlings was scored for the segregation of 95 AFLP and 22 SSR markers and a linkage map was constructed that covers a total genetic distance of 505 cM over seven linkage groups. The average linkage group length was 72.2 cM and there was an average of 17 markers per linkage group, of which at least two were codominant SSRs, allowing comparisons with previously published maps of raspberry. The two phenotypic traits, *A*_1 _and *dw*, mapped to linkage groups 3 and 6 respectively.

**Conclusion:**

The mapping of *A*_1 _will facilitate the discrimination of resistance genes from different sources and the pyramiding of aphid resistance genes in new raspberry cultivars; the mapping of *dw *will allow further investigations into the genetics of dwarfing habit in *Rubus*.

## Background

Red raspberry (*Rubus idaeus *L.) is an economically important member of the Rosaceae, cultivated mainly in Europe, the former USSR, and North and South America for its high value berries that are used both as dessert fruit and in processing. *Rubus idaeus *belongs to the sub-family Rosoideae and is a highly heterozygous diploid perennial species with a base chromosome number of seven (2*n *= 2*x *= 14).

Raspberry breeding programmes worldwide aim to produce improved cultivars to satisfy market demands which can also produce a profit for the growers. From making a controlled cross to naming a new cultivar takes between 10 and 15 years and there are many targets, including fruit quality, yield and season, as well as pest and disease resistance and plant habit. The fresh market demands perfect fruit with no pesticide residues and the number of pesticides available for horticultural crops is diminishing; so pest and disease resistance is increasingly important. The large raspberry aphid, *Amphorophora idaei*, transmits several viruses including Raspberry leaf mottle virus (RLMV), Raspberry leaf spot virus (RLSV), Black raspberry necrosis virus (BRNV) and *Rubus *yellow net virus (RYNV), and vector resistance has been central to the breeding programme at East Malling Research (EMR) for 50 years. Raspberry canes can grow up to 3 m and training and tipping canes to a manageable height is expensive. Many years ago breeders at East Malling tried to produce self-supporting types, which could be grown without the traditional trellis system, using cane characteristics from *R. cockburnianus *and *R. crataegifolius *and dwarfing habit from *R. ideaus *[1]. Unfortunately the fruit quality and yield penalties of this approach made it uneconomic.

Nearly 60 major gene traits have been reported in *Rubus idaeus *[2] including resistances to different biotypes of *Amphorophora idaei *and dwarf types. Several linkages have been proposed and these are summarised in the bibliography of Knight et al. [3]. Knight and Keep [4] highlighted the advantages of using resistance to *A. idaei *as a means of controlling virus infection in raspberry as early as 1958 and elucidated the genetic control of such a resistance derived from 'Baumforth A', assigning it to a single dominant gene, *A*_1 _[5]. It is clear from both papers that this resistance is strongly dependent upon the *A. idaei *biotype used for the screening; *A*_1 _was found to provide resistance against colonisation from biotypes 1 and 3 but it was not useful against the then uncommon biotype 2. Resistance gene *A*_1 _is reportedly linked to *d*_5 _for frilly dwarf with a recombination fraction of 3% [5]. The North American cultivar 'Chief' was reported to be the donor of additional resistance genes (*A*_2 _– *A*_7_) by Knight et al [6] with *A*_2 _reported to provide resistance against biotype 2. This resistance could also be achieved by the combination of *A*_3 _with either *A*_1 _or *A*_4_. Briggs [7] reported a fourth *A. idaei *biotype and gave more detailed information about the different aphid biotypes and explained the genetics of the aphid-host interaction. Despite biotype 4 being very rare in the field and difficult to overwinter in culture for experimental inoculations, resistance was identified from *R. idaeus *subsp. *strigosus *(accession L518) and explained by genes *A*_8 _and *A*_9_, both also conferring resistance to biotypes 1, 2 and 3. This resistance was later attributed to *A*_*L*518 _and might be the same as *A*_10 _from *R. occidentalis *described by Keep and Knight [8]. A third gene conferring resistance to all four biotypes was subsequently identified (*A*_*K*4*a*_) from a German clone of *R. idaeus *subsp. *vulgatus *[9]. The widespread growing of cultivars carrying *A*_1 _resistance has imposed a strong selection pressure on aphid populations causing biotype 2 to become predominant. Small populations of biotype 1 can be found only in wild raspberries in remote locations and biotypes 3 and 4 may be extinct. In addition, an *A*_10 _resistance-breaking biotype has been reported [10].

The pyramiding of several aphid resistance genes in breeding lines in order to provide more robust and long-lasting resistances has been an objective at EMR for 40 years [11]; however it is currently impossible to determine which and how many resistance genes are carried by resistant selections. This inability is due to not only the complex pedigrees of the plant material and the unavailability of some aphid biotypes, but, most importantly, to the equivalent effect of the genes *A*_*L*518_, *A*_10 _and *A*_*K*4*a*_, which makes them phenotypically indistinguishable; indeed they may be synonymous. Molecular markers would be a key tool in differentiating reported genes, identifying their presence in modern hybrid material and in managing strategies for pyramiding.

Jennings [12] reported and depicted a dwarf phenotype which he attributed to the recessive gene *dw *and speculated that *dw *was linked with *H *and *T*, the genes for pubescent stems and for presence or absence of anthocyanin. Soon afterwards, Keep [13] described sturdy dwarf, identical to that of Jennings [12], and four new dwarf phenotypes (crumpled, mottled, spidery and spindly) to add to the previously reported frilly [4]. She ascribed digenic control to the sturdy dwarf phenotype involving gene *d*_1 _and *d*_2 _and accepted Jennings's hypothesis of linkage between this character and the *H *and *T *genes.

With a view to marker-assisted breeding and map-based gene cloning, there is interest in extending linkage maps to include molecular markers. AFLPs [14] have become a useful tool for generating maps of large numbers of dominant markers without prior knowledge of DNA sequence [15], although their subsequent application as markers for selection of useful traits is limited to the breeding lines in which they were generated, unless they are first converted to SCARs [16]. Owing to their codominant nature and ease of transferability between germplasm, microsatellite markers (simple sequence repeats; SSRs), have found utility for a variety of purposes including the development of transferable, saturated linkage maps in many rosaceous genera [17-19], and SSRs have recently been developed for *Rubus *[20-22].

Graham et al. [23,24] employed both SSR and AFLP markers to produce a molecular linkage map of red raspberry (*R. ideaus*) based on a cross between the North American cultivar 'Latham' and the European cultivar 'Glen Moy' (L × GM). The most recent version of this map [24] is composed of 349 markers (69 SSR, 280 AFLP), covering 636 cM in eight linkage groups (six associated with both parents, two associated with one parent each). The L × GM map has been used to map the major gene *H *which determines cane pubescence to linkage group 2 of the *Rubus *genome and also to confirm the close association of that gene with resistance to cane botrytis (*Botrytis cinerea*), spur blight (*Didymella applanata*) and cane blight (*Leptosphaeria coniothyrium*), which were first reported by Knight and Keep [4], Jennings [25] and Jennings [26], respectively. In addition, a number of other QTL associated with disease resistance and plant morphology have been located on the L × GM map.

In this paper we report the analysis of an F_1 _population from a cross between two *R. idaeus *cultivars, 'Malling Jewel' and 'Malling Orion' raised as one of a series to investigate the distinctiveness of the different aphid resistance genes that have been used in the breeding programme at East Malling. Our aim was to map two agronomic characters, resistance to *A. idaei *infestation (*A*_1_) and dwarfing habit (*dw*), both of which segregate in this progeny, but have not previously been mapped in raspberry. We produced a framework map from the progeny using AFLP and SSR markers which provides coverage of the seven *Rubus *linkage groups previously defined by Graham et al. [24] and we successfully located the two phenotypic characters to discrete positions on two of the linkage groups defined using molecular markers.

## Results

### Phenotypic evaluation

In total, 146 seedlings were raised, of which 133 survived to be screened for resistance to *A. idaei *(biotype 1). On the 66 seedlings susceptible to *A. idaei *(*a*_1_*a*_1_), well-established colonies were visible a week after inoculation. Of these plants, 56 were normal and 10 were dwarfs. Sixty-two seedlings had no aphids or very few individuals, not constituting a colony, and were classed as resistant (*A*_1_*a*_1_). Of these, 50 were normal and 12 were dwarf. Five dwarf seedlings were too weak for inoculation and remained of unknown *A*_1 _status. Segregation for dwarf habit became apparent soon after germination, 117 being normal and 29 (sturdy) dwarfs; this accords with the 3:1 segregation ratio expected in a progeny from a cross of two heterozygotes segregating for a recessive character (χ^2 ^= 0.7). The mapping progeny is made up of 10 dwarf and 39 normal seedlings susceptible to *A. idaei *(biotype 1), and 10 dwarf and 35 normal seedlings resistant to *A. idaei *(biotype 1).

### Molecular marker segregation

In total, 94 seedlings from the mapping progeny (MJ × MO) were genotyped with molecular markers for map construction. From the 24 AFLP primer combinations tested, a total of 114 segregating products were scored in the parents of the progeny. Forty-five dominant markers segregated in 'M. Jewel' and 47 dominant markers segregated in 'M. Orion', whilst 22 markers segregated in both. Of the 52 SSR markers tested, a total of 22 polymorphic segregating markers were scored in the progeny. Three SSRs segregated in 'M. Jewel', seven segregated in 'M. Orion', and the remaining 12 segregated in both parents. As explained in the next section, 120 of the marker loci were mapped and significant deviation from the expected 1:1 and 3:1 ratios was detected at 25 of these. A total of 118 of the 136 molecular marker loci that segregated and the two morphological traits scored in the mapping progeny were used to construct a linkage map. The mapped markers comprised 29 AFLP loci segregating only in 'M. Jewel', 45 AFLP loci segregating only in 'M. Orion' and 22 AFLP segregating in both parents, along with all 22 SSR markers.

### Mapping in M. Jewel × M. Orion (MJ × MO)

**Figure 1:**
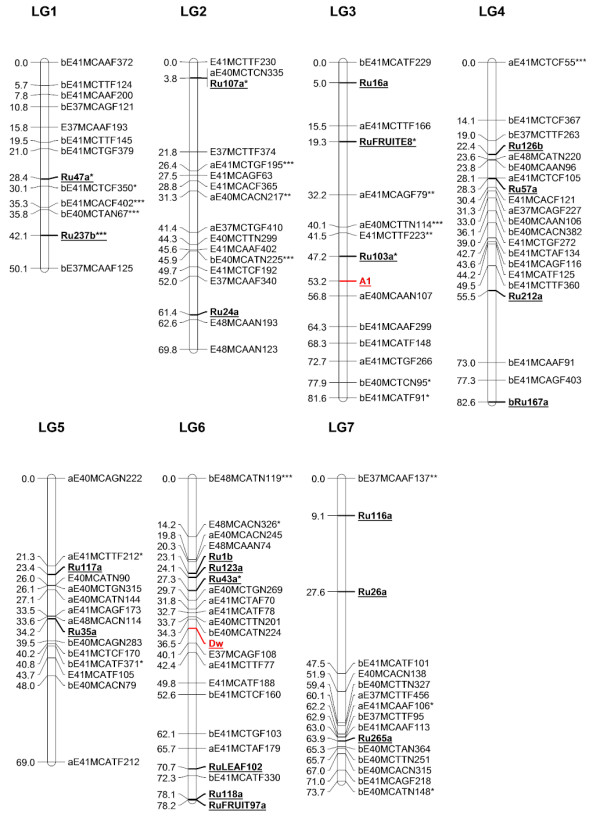
**A genetic linkage map of the MJ × MO raspberry mapping population**. A genetic linkage map of red raspberry from a cross of 'M. Jewel' × 'M. Orion' constructed from 22 microsatellites (SSRs) selected from the L × GM *Rubus *reference map (Graham et al 2006) and 95 AFLP markers. The map locates two phenotypic traits, resistance to the aphid *Amphorophora idaei *(*A*_1_) and dwarfing habit (*dw*). Mapping distances are given in centiMorgans (cM). Markers with segregation ratios deviating significantly from the expected ratios (P =< 0.05, 0.01, 0.001) are indicated with one, two or three asterisks respectively.

The 118 molecular markers and the two phenotypic traits, resistance to *A. idaei *(*A*_1_) and dwarfing habit (*dw*), located to seven linkage groups (LG), in accordance with the base chromosome number of *Rubus*, generally at a LOD of 5.0 or above (Figure 1). The exceptions to this were two regions that were associated with other markers, but at a LOD of just 2.0; LG4 markers bE41MCAAF91-bRu167a, and LG7 Ru26a-bE37MCAAF137. A further 13 AFLP loci that segregated in 'M. Jewel' were associated with LG1 and LG5; however, they remained unlinked when markers were assigned map positions, presumably because insufficient codominant markers segregated within these groups. The remaining five AFLP loci were not associated with any of the seven linkage groups revealed after linkage analysis. The *A*_1 _gene mapped to LG3, flanked by the codominant SSR Ru103a and the AFLP aE40MCAAN107, whilst the *dw *gene mapped to LG6, flanked by the AFLP markers bE40MCATN224 and E37MCAGF108. The 162 nt allele of the Ru103a marker was in coupling with the resistance allele *A*_1_. The closest SSRs to the *dw *gene were Ru43a and RuLEAF102 which flanked the gene at 9.2 cM and 34.2 cM respectively. Figure 1 shows the 120 marker loci that were mapped in MJ × MO, locating to seven discrete linkage groups (LG). Markers with segregation ratios deviating significantly from the expected ratios (P ≤ 0.05, 0.01, 0.001) are indicated with one, two or three asterisks respectively.

The map covers a total distance of 505 cM. The average length of the seven linkage groups was 72.2 cM, with an average of 17 markers per linkage group. Linkage group numbering follows that of Graham et al [24]. Linkage group 4 was the longest, with a total length of 82.6 cM, whilst LG1 was the shortest, at 50.1 cM. An interactive version of the MJ × MO map together with a table of segregation data, chi-squared values for goodness-of-fit to the expected Mendelian segregation ratios for the mapped markers and the linkage groups to which each marker is located have been placed in the Genome Database for Rosaceae [27].

## Discussion

The linkage map of the cross between *Rubus idaeus *'M. Jewel' × 'M. Orion' (MJ × MO) spans 505 cM and is composed of SSR and AFLP markers in seven linkage groups which have been numbered in accord with Graham et al. [24]. We have used this map to identify the locations of two previously unmapped phenotypic traits in *Rubus*, resistance to *Amphorophora idaei *(*A*_1_) and dwarfing habit (*dw*).

### Genome coverage and comparison with other Rubus linkage maps

**Figure 2:**
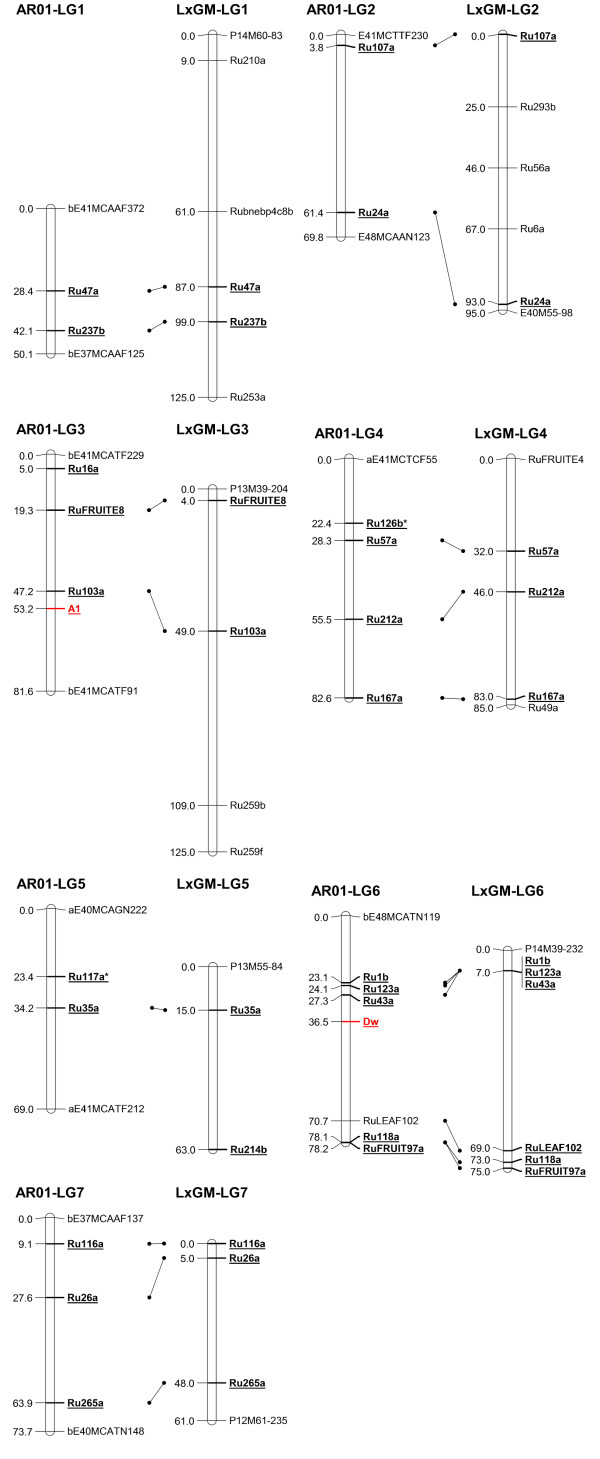
**Alignment of the MJ × MO and L × GM raspberry linkage maps**. Alignment of the 'M. Jewel' × 'M. Orion' and 'Latham' × 'GlenMoy' maps detailing markers mapped in common in both progenies. For clarity, markers defining the ends of the seven linkage groups have been included.

The linkage map of MJ × MO is significantly shorter than the 636 cM of the L × GM map of Graham et al. [24]. However, through the mapping of common SSR markers in both progenies, we have been able to compare the genetic regions covered by the two maps. Figure 2 compares the MJ × MO and L × GM maps with the locations of common markers highlighted. Linkage groups 2, 4, 6 and 7 of the MJ × MO map cover approximately the same genetic region as the L × GM map. Linkage group 5 of MJ × MO has just one mapped SSR common to the L × GM map, but covers a genetic distance of approximately the same size, whilst LG1 and LG3 are shorter than those of the L × GM map, covering 50 cM (compared with 125 cM) and 81 cM (compared with 125 cM) respectively. Thus we conclude that the MJ × MO genetic linkage map covers approximately 80% of the raspberry genome, with only partial regions of LG1 and LG3 not covered. Linkage group 1, however, is composed almost entirely of markers segregating only in 'M. Orion', with only one AFLP locus segregating in both parents.

### Aphid resistance

The map position of the major gene determining resistance to *A. idaei *in 'M. Orion' (*A*_1_) was located on LG3 of the MJ × MO map at approximately 5 cM from a codominant SSR marker Ru103a that segregates in both parents of the mapping population. We are not proposing that Ru103a be used directly for preselection for aphid resistance; for large scale selection, inoculation under glasshouse conditions remains cost-effective. However, now that an association between the *A*_1 _gene and a transferable codominant molecular marker has been established, we could screen progenies segregating for other aphid resistance genes with Ru103a to establish if any of these are synonymous with *A*_1 _or linked to it.

Selection pressure on the aphid has increased in the last 20 years as a result of the predominance of resistant cultivars. Resistance-breaking biotypes of *A. idaei *have been recorded on 'Autumn Bliss' which carries *A*_10 _but not on 'M. Leo' which carries both *A*_1 _and *A*_10_; this could be a consequence of gene pyramiding in the latter. In order to pyramid more resistance genes, especially those conferring resistance to biotypes 1 – 4, we need to establish whether or not the reported resistances are allelic. If different loci are responsible for the different reported sources of resistance, we will need appropriate markers in order to establish the genetic composition (number of resistance genes and level of homozygosity) of parental lines and resistant progenies. If the different reported resistances are allelic, only two forms of resistance could be pyramided into a single variety.

Interestingly, the region of LG3 associated with *A*_1 _is also the location of QTL for resistance to a number of *Rubus *pathogens, cane botrytis, spur blight and rust, on the map of Graham et al. [24].

### Dwarfing

There is a discrepancy between the single gene control for dwarf proposed by Jennings [12] and the two gene system proposed by Keep [13]. However, the 3:1 segregation observed in 'M. Jewel' × 'M. Orion' and the successful mapping of the character indicates that just one gene segregated in this progeny, consistent with the cross *Dwdw *× *Dwdw *according to the Jennings model or *D*_1_*D*_1_*D*_2_*d*_2 _× *d*_1_*d*_1_*D*_1_*d*_2 _or vice versa according to the Keep model. It should be noted however that the Keep model is idiosyncratic as *D*_1_*d*_1_*d*_2_*d*_2 _and *d*_1_*d*_1_*d*_2_*d*_2 _classes are assumed to be dwarf whilst *d*_1_*d*_1_*D*_2_*d*_2 _are normal. Why *D*_1_*d*_1_*d*_2_*d*_2 _should be dwarf is not explained.

In any case, if dwarf seedlings in the progeny from MJ × MO have the genotype *D*_1_*d*_1_*d*_2_*d*_2 _then intercrossing two such seedlings should give a progeny segregating for 1 normal (*D*_1_*D*_1_*d*_2_*d*_2_) to 3 dwarf and this could allow *d*_1 _to be mapped. As *dw *maps to LG6 and *H *(cane pubescence) maps to LG2 [24] they are unlinked, despite the speculation of Jennings [12] to the contrary. Keep [13] proposed the sturdy dwarf phenotype could have some interest for self-supporting breeding lines; however, because of other agronomic disadvantages such as reduced fertility and longevity this has not been pursued.

## Conclusion

Here we have produced a genetic linkage map from an F_1 _cross between two traditional red raspberry varieties using SSR and AFLP markers which have provided good coverage of the *Rubus *genome. We have mapped aphid resistance (*A*_1_) and sturdy dwarf habit (*dw*) for the first time, two single-gene traits that are of interest in raspberry breeding. For aphid resistance, we have identified a closely-linked codominant SSR marker that should be useful in discriminating aphid resistance genes from different sources and facilitate pyramiding.

## Methods

### Mapping population, phenotypic characters and DNA extraction

An F_1 _cross was made between two East Malling *R. idaeus *cultivars, 'M. Jewel' × 'M. Orion' (MJ × MO), and a total of 146 seedlings were raised, of which 133 survived to be scored phenotypically.

Individually potted seedlings were inoculated with four apterous adults of *A. idaei *biotype 1, 14 weeks after germination, and scored for colonisation (presence of both adults and nymphs) 10 days later. Seedlings with very little aphid infestation were re-inoculated with a fifth adult and re-assessed seven days later. Seedlings were also scored as (sturdy) dwarfs or normal.

A mapping progeny of 94 individuals was chosen, maintaining the segregation ratios for phenotypic traits observed in the original seedling population. Genomic DNA was extracted from the parents and the 94 seedlings following a scaled-down CTAB extraction [28] incorporating the addition of 1% [v/v] β-mercaptoethanol and 2% [w/v] polyvinyl pyrollidone (PVP 40) to the extraction buffer. The 94 seedlings were analysed with markers to generate data for linkage map construction.

### AFLP markers

Template DNA of the parents and seedlings was prepared for generation of AFLP fragments using AFLP analysis system II (Invitrogen) according to the manufacturer's protocol. Briefly, 125 ng genomic DNA was digested with *Eco*RI/*Mse*I. Pre-amplification was performed using the core AFLP primers E00 (5'GACTGCGTACATCCAG) and M00 (5'GATGAGTCCTGAGTAA), followed by selective amplification using primers with three base-pair extensions. Forward primers E37, E40, E41 and E48 were labelled using either 6-FAM or NED fluorescent dyes (Applied Biosystems) and used in combination with reverse primers M-CAA, M-CAC, M-CAG, M-CAT, M-CTA, M-CTC, M-CTG and M-CTT resulting in 24 different primer combinations. All PCR reactions were performed in a final volume of 20 μl, and 1 μl of the undiluted PCR was used for multiplex genotyping on an ABI3100 prism genetic analyser (Applied Biosystems). Data generated by capillary electrophoresis were collected and analysed using the GENESCAN and GENOTYPER (Applied Biosystems) software. Markers were named according to the primer combination used, the dye label of the forward primer and the size of the allele generated, i.e. bE37MCAGF227 was generated with primers E-37 labelled with 6-FAM and M-CAG and the allele generated was 227 nt in length. The prefix a or b indicates whether the marker segregated in the female (a) or male (b) parent. Those markers without a prefix segregated in both parents.

### Microsatellite markers

The primer pairs of the 52 previously-mapped SSR markers of Graham et al [24], were used to amplify DNA from the 'M. Jewel' and 'M. Orion' parental lines following the touchdown protocol of Sargent et al [29], with an annealing temperature between 55°C and 50°C, using a total of 1 ng of template DNA in a final reaction volume of 12.5 μl. The PCR products were analysed by electrophoresis at 75 V for 1 h 30 min through an EL800 Spreadex gel (Elchrom) which was subsequently stained for 30 min using SYBR gold nucleic acid stain (Invitrogen) to assess polymorphism and heterozygosity and therefore likely segregation in the MJ × MO population. A set of 23 heterozygous markers that mapped to seven linkage groups of the 'Latham' × 'Glen Moy' (L × GM) map of Graham et al [24] were chosen to define linkage groups identified in this study and the forward primers were tagged with an M13 pigtail [30].

PCR was performed for the chosen markers on genomic DNA from all 94 seedlings using the methods of Fukatsu et al. [30] with minor modifications. Briefly, PCRs were performed in a final reaction volume of 12.5 μl comprising 1 ng template DNA, 1 × PCR buffer, 1.5 mM Mg^2+^, 200 μM dNTPs, 0.2 μM reverse primer and 6-FAM labelled M13F primer (5' 6-FAMTGTAAAACGACGGCCAGT 3'), 0.008 μM M13-tagged forward primer (5' TGTAAAACGACGGCCAGT-PRIMER 3') and 0.25 U *Taq *polymerase (Invitrogen). Reactions were then carried out following the touchdown protocol described by Sargent et al. [29] between 55°C and 50°C. Products were visualised by electrophoresis on an ABI3100 prism genetic analyser (Applied Biosystems) and data generated were collected and analysed using the GENESCAN and GENOTYPER (Applied Biosystems) software and checked independently by two researchers.

### Data analysis and map construction

Chi-squared tests of goodness-of-fit to an expected segregation ratio of 1:1 or 3:1 were carried out for all markers segregating in the F_1 _progeny using JOINMAP 3.0 [31]. Linkage analysis was performed and markers were assimilated into groups for mapping with the application of the Kosambi mapping function. Marker positions were determined using a minimum LOD score threshold of 3.0, a recombination fraction threshold of 0.35, ripple value of 1.0, jump threshold of 3.0 and a triplet threshold of 5.0 and an integrated map of all segregating markers was constructed. The map presented was produced using MAPCHART for Windows [32].

## Authors' contributions

DJS was involved in the planning of the molecular experiments, generated the SSR and AFLP data, scored and analysed the segregation data and drafted the manuscript.

FF was involved in the planning of the glasshouse and molecular experiments, extracted DNA from the raspberry seedlings, scored and checked the AFLP data, carried out glasshouse aphid screenings and contributed to the manuscript.

AR extracted DNA from the raspberry seedlings, generated the AFLP and SSR data and carried out glasshouse aphid screenings.

VHK was involved in the planning of the glasshouse experiments, designed and performed the controlled crosses, carried out glasshouse aphid screenings, and contributed to the manuscript.

DWS was involved in the planning of the molecular experiments, scored and checked the SSR data and critically reviewed the manuscript.

KRT was involved in the planning of the glasshouse and molecular experiments, scored the SSR data and contributed to the manuscript.

All Authors read and approved the final manuscript.

## References

[B1] Knight RL, Keep E (1966). Breeding new soft fruits. Fruit – Present and Future.

[B2] Jennings DL (1988). Raspberries and Blackberries: Their breeding, diseases and growth.

[B3] Knight RL, Parker JH, Keep E (1972). Abstract Bibliography of Fruit Breeding and Genetics, 1956–1969, Rubus and Ribes.

[B4] Knight RL, Keep E (1958). Developments in soft fruit breeding at East Malling. Report of East Malling Research Station for 1957.

[B5] Knight RL, Keep E, Briggs JB (1959). Genetics of resistance to *Amphorophora rubi *(Kalt.) in the raspberry. I. The gene *A*_1 _from Baumforth A. Journal of Genetics.

[B6] Knight RL, Briggs JB, Keep E (1960). Genetics of resistance to *Amphorophora rubi *(Kalt.) in the raspberry. II. The gene *A*_2 _– *A*_7 _from the American variety, Chief. Genetical Research.

[B7] Briggs JB (1965). The distribution, abundance and genetic relationships of four strains of the rubus aphid (*Amphorophorarubi *(Kalt.)) in relation to raspberry breeding. The Journal of Horticultural Science.

[B8] Keep E, Knight RL (1967). A new gene from *Rubus occidentalis *L. for resistance to strains 1, 2, and 3 of the Rubus aphid, *Amphorophora rubi *Kalt. Euphytica.

[B9] Keep E, Knight RL, Parker JH (1970). Further data on resistance to the Rubus aphid *Amphorophora rubi *(Kltb.). Report of East Malling Research Station for 1969.

[B10] Birch ANE, Geoghegan IE, Majerus MEN, Hackett C, Allen J (1997). Interactions between plant resistance genes, pest aphid populations and beneficial aphid predators. SCRI Annual Report for 1996.

[B11] Keep E, Knight RL (1968). Use of the black raspberry (*Rubus occidentalis *L.) and other *Rubus *species in breeding red raspberries. Report of East Malling Research Station for 1967.

[B12] Jennings DL (1967). Balanced lethals and polymorphism in *Rubus idaeus*. Heredity.

[B13] Keep E (1968). Dwarfing in the raspberry, *Rubus idaeus *L. Euphytica.

[B14] Vos P, Hogers R, Bleeker M, Reijans M, van de Lee T, Hornes M, Frijters A, Pot J, Peleman J, Kuiper M, Zabeau M (1995). AFLP: A new technique for DNA fingerprinting. Nucleic Acids Research.

[B15] Lu ZX, Sosinski B, Reighard GL, Baird VW, Abbott AG (1998). Construction of a genetic linkage map and identification of AFLP markers for resistance to root-knot nematodes in peach rootstocks. Genome.

[B16] Evans KM, James CM (2003). Identification of SCAR markers linked to *Pl-w *mildew resistance in apple. Theoretical and Applied Genetics.

[B17] Aranzana MJ, Pineda A, Cosson P, Dirlewanger E, Ascasibar J, Cipriani G, Ryder CD, Testolin R, Abbot A, King GJ, Iezzoni AF, Arús P (2003). A set of simple sequence repeat (SSR) markers covering the *Prunus *genome. Theoretical and Applied Genetics.

[B18] Silfverberg-Dilworth E, Matasci C, Van de Weg E, Van Kaauwen MPW, Walser M, Kodde LP, Soglio V, Gianfranceschi L, Durel CE, Costa T, Yamamoto T, Koller B, Gessler C, Patocchi A (2006). Microsatellite markers spanning the apple (Malus × domestica Borkh.) genome. Tree Genetics and Genomics.

[B19] Sargent DJ, Clarke J, Simpson DW, Tobutt KR, Arús P, Monfort A, Vilanova S, Denoyes-Rothan B, Rousseau M, Folta KM, Bassil NV, Battey NH (2006). An enhanced microsatellite map of diploid *Fragaria *. Theoretical and Applied Genetics.

[B20] Amsellem L, Dutech C, Billotte N (2001). Isolation and characterization of polymorphic microsatellite loci in *Rubus alceifolius *Poir. (Rosaceae), an invasive weed in La Reunion island. Molecular Ecology Notes.

[B21] Graham J, Smith K, Woodhead M, Russell J (2002). Development and use of simple sequence repeat SSR markers in *Rubus *species. Molecular Ecology Notes.

[B22] Stafne ET, Clark JR, Weber CA, Graham J, Lewers KS (2005). Simple sequence repeat (SSR) markers for genetic mapping of raspberry and blackberry. Journal of the American Society for Horticultural Science.

[B23] Graham J, Smith K, MacKenzie K, Jorgenson L, Hackett C, Powell W (2004). The construction of a genetic linkage map of red raspberry (*Rubus idaeus *subsp *idaeus*) based on AFLPs, genomic-SSR and EST-SSR markers. Theoretical and Applied Genetics.

[B24] Graham J, Smith K, Tierney I, MacKenzie K, Hacket CA (2006). Mapping gene *H *controlling cane pubescence in raspberry and its association with resistance to cane botrytis and spur blight, rust and cane spot. Theoretical and Applied Genetics.

[B25] Jennings DL (1982). Resistance to *Didymellaapplanta *in red raspberry and some related species. Annals of Applied Biology.

[B26] Jennings DL (1982). Further evidence on the effect of gene *H *which confers cane hairiness, on resistance to raspberry diseases. Euphytica.

[B27] Jung S, Jesudurai C, Staton M, Du ZD, Ficklin S, Cho IH, Abbott A, Tomkins J, Main D (2004). GDR (Genome Database for Rosaceae): integrated web resources for Rosaceae genomics and genetics research. BMC Bioinformatics.

[B28] Doyle JJ, Doyle JL (1987). A rapid DNA isolation procedure for small quantities of fresh leaf tissue. Phytochemical Bulletin.

[B29] Sargent DJ, Hadonou AM, Simpson DW (2003). Development and characterisation of polymorphic microsatellite markers from *Fragaria viridis*, a wild diploid strawberry. Molecular Ecology Notes.

[B30] Fukatsu E, Isoda K, Hirao T, Takahashi M, Watanabe A (2005). Development and characterization of simple sequence repeat DNA markers for *Zelkova serrata*. Molecular Ecology Notes.

[B31] Van Ooijen JW, Voorrips RE (2001). JoinMap 3.0: Software for the calculation of genetic linkage maps.

[B32] Voorrips RE (2002). MapChart: Software for the graphical presentation of linkage maps and QTLs. Journal of Heredity.

